# Supplementary material for: Comparative genomics provides new insights into the diversity, physiology, and sexuality of the only industrially exploited tremellomycete: Phaffia rhodozyma
Source: BMC Genomics. 2016 Nov 9;17:901. doi: 10.1186/s12864-016-3244-7 (PMC5103461; doi:10.1186/s12864-016-3244-7)
Supplement: Additional file 6: — List of orphan genes with links to PFAM (related to Additional file 1: Table S1). (ZIP 1428 kb) [file 12864_2016_3244_MOESM6_ESM.zip › BLAST_HTML_FTR/G04269_P.html]

BLAST Search Results


```
BLASTP 2.2.27+


Reference:
Stephen F. Altschul, Thomas L. Madden, Alejandro A. Schäffer,
Jinghui Zhang, Zheng Zhang, Webb Miller, and David J. Lipman (1997),
"Gapped BLAST and PSI-BLAST: a new generation of protein database
search programs", Nucleic Acids Res. 25:3389-3402.


Reference for
composition-based statistics:
Alejandro A. Schäffer, L. Aravind, Thomas L. Madden, Sergei
Shavirin, John L. Spouge, Yuri I. Wolf, Eugene V. Koonin, and
Stephen F. Altschul (2001), "Improving the accuracy of PSI-BLAST
protein database searches with composition-based statistics and
other refinements", Nucleic Acids Res. 29:2994-3005.


Database: nr
           71,551,133 sequences; 26,053,659,533 total letters


Query= G04269_P

Length=1110
                                                                      Score     E
Sequences producing significant alignments:                          (Bits)  Value

emb|CED83684.1|  hypothetical protein [Xanthophyllomyces dendrorh...  2214    0.0  
gb|KIJ99383.1|  hypothetical protein K443DRAFT_174294 [Laccaria a...  42.0    5.0  


 >emb|CED83684.1| hypothetical protein [Xanthophyllomyces dendrorhous]
Length=1109

 Score = 2214 bits (5736),  Expect = 0.0, Method: Compositional matrix adjust.
 Identities = 1108/1109 (99%), Positives = 1109/1109 (100%), Gaps = 0/1109 (0%)

Query  1     MPSTATLSYIPVHMPDLQTSSATTKFHSHTSLSNSATLYPPLQSTSPSTSPTDRTDTGHI  60
             MPSTATLSYIPVHMPDLQTSSATTKFHSHTSLSNSATLYPPLQSTSPSTSPTDRTDTGHI
Sbjct  1     MPSTATLSYIPVHMPDLQTSSATTKFHSHTSLSNSATLYPPLQSTSPSTSPTDRTDTGHI  60

Query  61    RWTTDHQLEDGERYGRFTLKQNASGLANPASERSGSCDSGRTELSSTTGSSNSSDTSITS  120
             RWTTDHQLEDGERYGRFTLKQNASGLANPASERSGSCDSGRTELSSTTGSSNSSDTSITS
Sbjct  61    RWTTDHQLEDGERYGRFTLKQNASGLANPASERSGSCDSGRTELSSTTGSSNSSDTSITS  120

Query  121   TTSTAPSLASPASSIVQLKTTISPNLTNGQEPLSPSGKDKDLKTLRRSSSGFGMFKGWFS  180
             TTSTAPSLASPASSIVQLKTTISPNLTNGQEPLSPSGKDKDLKTLRRSSSGFGMFKGWFS
Sbjct  121   TTSTAPSLASPASSIVQLKTTISPNLTNGQEPLSPSGKDKDLKTLRRSSSGFGMFKGWFS  180

Query  181   GSSAGPKKVKEKGKPSGGKTSLQEGCLVMQTSGATGASQSSEFPPPSSSSVLPELVFPSP  240
             GSSAGPKKVKEKGKPSGGKTSLQEGCLVMQTSGATGASQSSEFPPPSSSSVLPELVFPSP
Sbjct  181   GSSAGPKKVKEKGKPSGGKTSLQEGCLVMQTSGATGASQSSEFPPPSSSSVLPELVFPSP  240

Query  241   AFMGQAGRRFLSDSPSPGGSPTGSPSFPPISLPSIPNIPSSSPSQSCPVRQDFGSSQSSI  300
             AFMGQAGRRFLSDSPSPGGSPTGSPSFPPISLPSIPNIPSSSPSQSCPVRQDFGSSQSSI
Sbjct  241   AFMGQAGRRFLSDSPSPGGSPTGSPSFPPISLPSIPNIPSSSPSQSCPVRQDFGSSQSSI  300

Query  301   QQQQQQPPTLTPLQSLHLLSSQKLSLLLRPPSPHPFFMSRLNPASGKDGETRRYPASTNP  360
             QQQQQQPPTLTPLQSLHLLSSQKLSLLLRPPSPHPFFMSRLNPASGKDGETRRYPASTNP
Sbjct  301   QQQQQQPPTLTPLQSLHLLSSQKLSLLLRPPSPHPFFMSRLNPASGKDGETRRYPASTNP  360

Query  361   VTAQSTPRRRVVGLTAEVGVRGLMHRLETDARLSADEMEALIRLSQARPVSLQRDKSGRL  420
             VTAQSTPRRRVVGLTAEVGVRGLMHRLETDARLSADEMEALIRLSQARPVSLQRDKSGRL
Sbjct  361   VTAQSTPRRRVVGLTAEVGVRGLMHRLETDARLSADEMEALIRLSQARPVSLQRDKSGRL  420

Query  421   IASISPTSDASVSTAEFAATSAVPRANLPLAGSGILAFISRPTLEVRSIVLVPAANDPDR  480
             IASISPTSDASVSTAEFAATSAVPRANLPLAGSGILAFISRPTLEVRSIVLVPAANDPDR
Sbjct  421   IASISPTSDASVSTAEFAATSAVPRANLPLAGSGILAFISRPTLEVRSIVLVPAANDPDR  480

Query  481   IVVEPILPARGLAVMELEFSEGMEAYAREVIKKSSASFEVNVPGTRHSTSGTTLPSLVNS  540
             I+VEPILPARGLAVMELEFSEGMEAYAREVIKKSSASFEVNVPGTRHSTSGTTLPSLVNS
Sbjct  481   IIVEPILPARGLAVMELEFSEGMEAYAREVIKKSSASFEVNVPGTRHSTSGTTLPSLVNS  540

Query  541   LVGRRSPTDPSPADGLKVVIDRVPTVRETVLPGIKLRRSSSTPSSPIKANASSEPVNEFL  600
             LVGRRSPTDPSPADGLKVVIDRVPTVRETVLPGIKLRRSSSTPSSPIKANASSEPVNEFL
Sbjct  541   LVGRRSPTDPSPADGLKVVIDRVPTVRETVLPGIKLRRSSSTPSSPIKANASSEPVNEFL  600

Query  601   SPTMNNVPHASKLKSSLSSSSLGLQKQQQQQQLAIPRSALSKPRRSMSSMNLVTRRSFPP  660
             SPTMNNVPHASKLKSSLSSSSLGLQKQQQQQQLAIPRSALSKPRRSMSSMNLVTRRSFPP
Sbjct  601   SPTMNNVPHASKLKSSLSSSSLGLQKQQQQQQLAIPRSALSKPRRSMSSMNLVTRRSFPP  660

Query  661   PVGSQFAESALEDDDKEDETPLSEIQRRHSTIALTPVPSQSTRARDRVRFAQVDVMEDDR  720
             PVGSQFAESALEDDDKEDETPLSEIQRRHSTIALTPVPSQSTRARDRVRFAQVDVMEDDR
Sbjct  661   PVGSQFAESALEDDDKEDETPLSEIQRRHSTIALTPVPSQSTRARDRVRFAQVDVMEDDR  720

Query  721   SCKEDLGKDLDRQGEAPWMDSQAEIRAREDAEWEALERDRLRADEEERRRQERYRAEVAA  780
             SCKEDLGKDLDRQGEAPWMDSQAEIRAREDAEWEALERDRLRADEEERRRQERYRAEVAA
Sbjct  721   SCKEDLGKDLDRQGEAPWMDSQAEIRAREDAEWEALERDRLRADEEERRRQERYRAEVAA  780

Query  781   ARERRDLAKLGSKKGNNGNYEEWGALSARVATSSRTTRSESLRTLGENSASPMDTNQPAA  840
             ARERRDLAKLGSKKGNNGNYEEWGALSARVATSSRTTRSESLRTLGENSASPMDTNQPAA
Sbjct  781   ARERRDLAKLGSKKGNNGNYEEWGALSARVATSSRTTRSESLRTLGENSASPMDTNQPAA  840

Query  841   RRGDRSSRVFAPVELSIHPDRSRQNALEISPSTSNSTTPSHSPQSGSPPSSSPRSSILSS  900
             RRGDRSSRVFAPVELSIHPDRSRQNALEISPSTSNSTTPSHSPQSGSPPSSSPRSSILSS
Sbjct  841   RRGDRSSRVFAPVELSIHPDRSRQNALEISPSTSNSTTPSHSPQSGSPPSSSPRSSILSS  900

Query  901   ARPSPVSTNSGGISKRHSTAAITPSPLSSSIENQPVRGSQQKSALSSPGAQPPVALSTSA  960
             ARPSPVSTNSGGISKRHSTAAITPSPLSSSIENQPVRGSQQKSALSSPGAQPPVALSTSA
Sbjct  901   ARPSPVSTNSGGISKRHSTAAITPSPLSSSIENQPVRGSQQKSALSSPGAQPPVALSTSA  960

Query  961   PSRPSSGHHSSLSLQIPPPYASSPMMVHSYSTPSLAGNTHYTHQPAYSPVLTSPPTTYYH  1020
             PSRPSSGHHSSLSLQIPPPYASSPMMVHSYSTPSLAGNTHYTHQPAYSPVLTSPPTTYYH
Sbjct  961   PSRPSSGHHSSLSLQIPPPYASSPMMVHSYSTPSLAGNTHYTHQPAYSPVLTSPPTTYYH  1020

Query  1021  PHHLHHVPQPQYYPQPAYSVQGYPFSTHYTQFGFPNQHQGFSPLMTPQYIQPHQAAYQQP  1080
             PHHLHHVPQPQYYPQPAYSVQGYPFSTHYTQFGFPNQHQGFSPLMTPQYIQPHQAAYQQP
Sbjct  1021  PHHLHHVPQPQYYPQPAYSVQGYPFSTHYTQFGFPNQHQGFSPLMTPQYIQPHQAAYQQP  1080

Query  1081  IHIQPQPHMGSRSSRANLRPTPGSSRQER  1109
             IHIQPQPHMGSRSSRANLRPTPGSSRQER
Sbjct  1081  IHIQPQPHMGSRSSRANLRPTPGSSRQER  1109


>gb|KIJ99383.1| hypothetical protein K443DRAFT_174294 [Laccaria amethystina LaAM-08-1]
Length=813

 Score = 42.0 bits (97),  Expect = 5.0, Method: Compositional matrix adjust.
 Identities = 34/93 (37%), Positives = 48/93 (52%), Gaps = 11/93 (12%)

Query  708  VRFAQVDVMEDDRSCKEDLGKDLDRQGEAPWMDSQAEIRAREDA------EWEALERDRL  761
            VRFA+ D   DD      +     R+G+A ++  +   RARE+A      E EALERDRL
Sbjct  421  VRFAEDD--SDDEIPLHIVRMKKKREGKAKFLRREQLKRAREEADEKRRLEEEALERDRL  478

Query  762  R---ADEEERRRQERYRAEVAAARERRDLAKLG  791
            R     E+  R +  +  E+AA R RR+  + G
Sbjct  479  RLAKEGEKRERERRLFAEELAATRMRREFQRAG  511


 Score = 41.6 bits (96),  Expect = 6.5, Method: Compositional matrix adjust.
 Identities = 53/197 (27%), Positives = 81/197 (41%), Gaps = 47/197 (24%)

Query  319  LSSQKLSLLLR----PPSP-HPFFMSRLNPASGKDGETRRYPASTNPVTAQSTPRRRVVG  373
            L+ + L +L++    PP P  PF    L P          YP S+N    +  PRR  + 
Sbjct  171  LARKNLVVLVKNSLAPPLPISPFVQHSLGPI---------YPRSSN--QPRVLPRR--LP  217

Query  374  LTAEVGVRGLMHRLETDARLSADEMEALIRLSQARP----VSLQRDKSGRLIASISPTSD  429
            ++A +  + L+ RLE  + +     E+ +    +RP    VSLQR               
Sbjct  218  MSAVMMKKRLLTRLEDSSFIFPSSEESFLHYFNSRPTSSEVSLQR---------------  262

Query  430  ASVSTAEFAATSAVPRANLPLAGSGILAFISRPTLEVRSIVLVPAANDPD-RIVVEPILP  488
                T  F  +       +  +  G+  +ISRP  E R +V VP  ND   R V  P   
Sbjct  263  ----TLTFDTSYPERTTRIFRSSPGLRRWISRPCFEDRFVVFVPEGNDVKCRAVSGP---  315

Query  489  ARGLAVMELEFSEGMEA  505
               LA+  LE+SE ++ 
Sbjct  316  --SLAIAALEYSEYLDV  330


Lambda      K        H        a         alpha
   0.309    0.124    0.352    0.792     4.96 

Gapped
Lambda      K        H        a         alpha    sigma
   0.267   0.0410    0.140     1.90     42.6     43.6 

Effective search space used: 13710332435676


  Database: nr
    Posted date:  Sep 23, 2015 12:05 AM
  Number of letters in database: 26,053,659,533
  Number of sequences in database:  71,551,133


Matrix: BLOSUM62
Gap Penalties: Existence: 11, Extension: 1
Neighboring words threshold: 11
Window for multiple hits: 40
```
